# Exploring the attitudes of general medical students toward older adult’s care in a lower middle-income country: implications for medical education

**DOI:** 10.1186/s12909-023-04626-1

**Published:** 2023-09-08

**Authors:** Marzieh Nojomi, Salime Goharinezhad, Rasoul Saraei, Saeideh Goharinejad, Ghobad Ramezani, Maryam Aalaa

**Affiliations:** 1 Preventive Medicine and Public Health Research Center, Psychosocial Health Research Institute, Department of Community and Family Medicine, School of Medicine, Iran University of Medical Sciences, Tehran, Iran; 2 Department of Health Services Management, School of Health Management and Information Sciences, Iran University of Medical Sciences, Tehran, Iran; 3 Student Research Committee, Iran University of Medical Sciences, Tehran, Iran; 4 Faculty of Healthcare Science, Deggendorf Institute of Technology, Pfarrkirchen, Germany; 5 Education Development Center, Kermanshah University of Medical Sciences, Kermanshah, Iran; 6 Center for Educational Research in Medical Sciences (CERMS), Department of Medical Education, School of Medicine, Iran University of Medical Sciences (IUMS), Tehran, Iran; 7 Department of e-Learning in Medical Education, Center of Excellence for e-Learning in Medical Education, School of Medicine, Tehran University of Medical Sciences, Tehran, Iran

**Keywords:** Older adult care, General Medical Students, Attitudes, Medical Education

## Abstract

**Objective:**

The motivation for this study stemmed from the growing population of older adults and the increasing demand for healthcare professionals who possess the necessary skills and knowledge to provide quality care to this demographic. By exploring the attitudes, perceptions, and beliefs of medical students towards older adult care, the study aimed to identify gaps in their training and areas where improvements can be made to better equip them for this critical aspect of healthcare.

**Method:**

This study was a qualitative thematic analysis. The participants of this research were selected from among the general medicine internship students of Iran University of Medical Sciences by purposive sampling method. In-depth individual semi-structured interviews were used to collect data. Sampling continued until data saturation. The interviews were recorded, transcribed, and analyzed using a hybrid approach of inductive and deductive thematic analysis. Using this approach, the analysis of the data became more adaptable and open-ended, free from the restrictions of pre-existing theoretical frameworks. MAXQDA 22 was used to analyze qualitative data.

**Results:**

A total of 27 medical students were interviewed semi-structured, and audio files were transcribed immediately after the interview. In the process of reading the interviews carefully and separating the conceptual units into codes, 167 primary codes were obtained, and these codes were divided into five main categories under the title of root factors after constant comparison analysis. Five main themes are including discrimination in service delivery, a lack of inter-professional training, interpersonal communication skills, inadequate infrastructure and human resources, and enhancing attitudes towards older person care through experiential learning.

**Conclusion:**

This study sheds light on the attitudes and perceptions of medical students toward older adult care in a lower-middle-income country. The findings reveal that there are significant gaps in their training and preparation for providing quality care to this demographic profile. The four main categories identified as root factors highlight key areas where improvements can be made in medical education. It is recommended that medical schools in low and middle-income countries consider incorporating these themes into their curricula to better equip future healthcare professionals with the necessary skills and knowledge to provide quality care to older adults.

## Introduction

The global population is aging rapidly, with the number of individuals aged 60 years and older projected to reach 2.1 billion by 2050 [1]. This demographic shift has significant implications for healthcare systems around the world, as older adults often require more complex care and support than younger patients due to age-related decline in physical and cognitive function [2]. In particular, caring for older adults with multiple chronic conditions can be challenging and requires specialized knowledge, skills, and attitudes on the part of healthcare providers [3].

Despite the growing need for care for older adults, medical students may not always have positive attitudes towards caring for this population. Prior research has shown that negative attitudes towards older adults are common among medical students, residents, and practicing physicians [4]. These attitudes can manifest as ageism, which is defined as prejudice or discrimination based on age, and can lead to suboptimal care and negative health outcomes for older adults [5]. For example, ageist attitudes may result in physicians being less likely to screen older adults for cancer, provide appropriate pain management, or refer them for rehabilitation services [6].

To address these issues, it is important to understand the underlying factors that contribute to these negative attitudes among medical students. Most studies of medical students’ attitudes towards older adult care have been conducted in high-income countries (HICs) such as the United States, Canada, and Australia [7] and there is a lack of research on medical students’ attitudes towards older adult care in lower middle-income countries, which may not be generalizable to other contexts. Therefore, there is an evidence gap regarding medical students’ attitudes towards older adult care in lower middle-income countries.

On the other hand, the challenges and opportunities associated with caring for older adults differ across different cultural and socioeconomic contexts. Therefore, it is important to conduct research on medical students’ attitudes towards older adult care in a range of settings to gain a more comprehensive understanding of this issue.

This qualitative study aims to explore the attitudes of general medical students towards older adult care in a lower middle-income country, Iran. The study is guided by the theoretical and conceptual framework of medical education and motivation to general medicine in international contexts. The research questions guiding this study are:

What are the attitudes of general medical students towards older adult care in Iran? What are the underlying factors that contribute to these attitudes? And what potential strategies can be identified for addressing negative attitudes towards older adult care among medical students in Iran?

By conducting in-depth interviews with medical students, we aim to gain insights into their attitudes towards older adult care and identify potential strategies for addressing negative attitudes. This research could inform efforts to improve medical education and training related to caring for older adult patients in Iran and other lower middle-income countries.

## Methods

### Study design

The study design used in this research was qualitative and descriptive-interpretive. This methodology is appropriate when the aim of the study is to explore a phenomenon and consider its importance, especially if there is insufficient existing knowledge in that field. The research employed in-depth individual semi-structured interviews to collect data. This study’s method was guided by the Consolidated Criteria for Reporting Qualitative Research (COREQ) checklist for reporting qualitative studies [8].

### Study setting

Teaching hospitals in Iran are affiliated with medical universities. The country has a total of 68 medical universities that are geographically distributed across the nation. In the capital city, Tehran, there are three prominent universities that cater to the healthcare needs of over 13 million people. These three universities, collectively known as the University of Medical Sciences, are responsible for providing comprehensive health services in the region. Among these universities, the Iran University of Medical Sciences (IUMS) serves the largest population in the Tehran Province, specifically overseeing healthcare delivery in the western regions that comprise more than 5 million individuals. IUMS operates 12 teaching hospitals that play a crucial role in clinical education for medical and health profession students during their clerkship. These teaching hospitals primarily function as secondary and tertiary care centers, offering valuable learning opportunities for students to gain hands-on experience and delve into complex medical cases.

The study was conducted at the Iran University of Medical Sciences and its affiliated teaching hospitals, where medical students completed their clinical practice as part of their medicine internship.

Over the course of ten months, starting in May 2021 and ending in March 2022, interviews were conducted with participants either in-person or via phone, based on their stated preference. For in-person data collection, a private room was provided at the teaching hospitals affiliated with the Iran University of Medical Sciences. Prior to the interviews, participants were informed about the interviewers’ background and qualifications.

### Sampling and recruitment of participants

Purposive sampling method was used to select participants from among the general medicine internship students at Iran University of Medical Sciences. Sampling continued until data saturation was achieved. In other words, the researchers kept selecting participants and conducting interviews until no new insights or themes emerged from the data. A total of 27 participants included in the study. The researchers approached potential participants in-person or via email after obtaining approval from the university.

### Data collection

Data was collected through in-depth individual semi-structured interviews. The interview guide (Table 1) was pre-tested with two medical education professionals and two undergraduate medical students and consisted of three sections. The first two sections aimed to explore participants’ attitudes towards older adult care and the factors that influenced these attitudes. The third section focused on participants’ explanations for addressing negative attitudes.

The interviews were conducted by two interviewers (SG, GHR), who had experience conducting qualitative research and were trained in interviewing techniques and Both interviewers held Ph.D. degrees in health sciences. The researchers established professional relationships with the participants through the informed consent process and by introducing themselves as researchers.

Over the course of ten months, starting in May 2021 and ending in March 2022, interviews were conducted with participants either in-person or via phone, based on their stated preference.

Audio recording was used to capture the interviews, which were transcribed verbatim. Field notes were taken during the interviews. The duration of each interview was between 30 and 40 min.

**Table 1:** Interview guide questions

Section	Interview Guide
1. Attitudes Towards Older Adult Care	Asking open-ended questions to explore the attitudes of medical students towards older adult care in Iran. • What are your general perceptions about providing healthcare to older adults in Iran? • How do you feel about working with older adult patients compared to younger patients? • Do you think there are any specific challenges or benefits associated with caring for older adults? If yes, please explain. • Have you had any personal experiences that have influenced your attitudes towards older adult care?
2. Factors Influencing Attitudes	Probe deeper into the underlying factors that contribute to the attitudes identified in the previous section. • What are some of the factors that shape your attitudes towards older adult care? • Are there any cultural, societal, or educational factors that influence your perception of older adults and their care? • How does the current medical curriculum address the care of older adults? Do you think it adequately prepares you? • Are there any personal or professional role models who have influenced your perception of older adult care? If yes, how?
3. Addressing Negative Attitudes	Explore potential strategies for addressing negative attitudes towards older adult care among medical students in Iran. • In your opinion, what can be done to improve medical education regarding the care of older adults? • Are there any specific training programs or interventions that you think would be effective in changing negative attitudes? • How can the curriculum be modified to enhance awareness, empathy, and skills related to older adult care? • What kind of support systems or resources do you believe would be helpful for medical students to develop positive attitudes?

### Data analysis

In this study, 27 medical students were interviewed, and the resulting 18 h and 38 min of audio material were transcribed for analysis. The analysis was conducted in Persian by the principal investigator (SG), along with a research team (RS, SG) and Medical education consulting (MA, GHR), using MAXQDA 22 software. For the thematic analysis of the data in this study, both inductive and deductive labeling of themes were used in a hybrid approach. This approach allowed for a more flexible and open-ended analysis of the data, without being constrained by pre-existing theoretical frameworks [9].

The transcripts and recordings were carefully reviewed to familiarize the team with the data, and the principle investigator performed line-by-line coding and extensive note-taking to engage with the data on an abstract level. The codes were then merged into overarching themes and subthemes by two researchers (RS, SG) through an iterative process. The team examined the codes and data excerpts to identify patterns and consolidate them into candidate themes, which were reviewed to ensure coherence and distinctiveness. The final phase of analysis involved naming the themes, creating descriptions, and selecting interview excerpts for presentation. The supporting quotes have been translated into English and are accompanied by anonymous participant identifiers. To ensure the trustworthiness of the interview data, various measures were taken according to the Table 2.

**Table 2:** Measures for ensuring trustworthiness

Trustworthiness Measures	Description
Credibility	• Multiple researchers reviewed and analyzed the data to ensure the accuracy and consistency of the findings. • The research team used a hybrid approach to thematic analysis, which allowed for flexibility and openness to the data.
Transferability	• The study sample was selected from a range of medical students in a lower middle-income country, which enhances the transferability of the findings to similar settings and contexts.
Dependability	• A clear and systematic approach was used for data collection and analysis, which was documented in detail in the study. • The research team maintained an audit trail of the research process, including notes and records of decision-making.
Confirmability	• The research team used MAXQDA software to manage and code the data, which increases the transparency and objectivity of the analysis. • The research team engaged in ongoing reflexivity and critical reflection throughout the research process to minimize bias and increase the credibility of the findings.

### Ethical consideration

The Research Ethics Committee of Iran University of Medical Sciences approved the study under code IR.IUMS.REC.1399.1115, and all ethical considerations were taken into account throughout the research process.

## Results

The study involved conducting semi-structured interviews with 27 medical students, followed by immediate implementation of audio files. After a thorough analysis of the transcripts, 167 primary codes were identified and grouped into five main categories under the title “Root Factors Affecting the Attitudes of Medical Students towards older adult Care”. These categories included discrimination in service delivery, lack of cooperative and interprofessional training, interpersonal interactions and communication, Limited Knowledge, Human Resources, and Physical Infrastructure in older adults’ care and Enhancing Attitudes Towards older person Care through Experiential Learning. Table 3 indicates the main theme and sub-themes.

**Table 3:** Themes, and subthemes emerging from the analysis

Theme	Sub-Theme
Discrimination in service delivery	Ageism and stereotyping
	Lack of cultural awareness or sensitivity to diverse populations
	Inadequate access to health care services
	Inadequate training regarding discrimination
Lack of interprofessional education	Limited opportunities for interdisciplinary education
	The need for more teamwork and leadership training
	Inadequate collaboration between health care professionals
	Lack of communication skills training
Interpersonal interactions and communications	Communication barriers
	Resistant patients and family members
	Need for more patient-centered care approaches
	Empathy and active listening skills
Lack of knowledge, human and physical infrastructure in older adult care	Inadequate training related to the geriatric medicine
	Lack of qualified health care professionals
	Age-friendly design or accessibility features
Enhancing Attitudes Towards older person Care through Experiential Learning	Hands-on experiences in the clinical settings
	Mentorship programs
	Community Engagement & home visitations
	Role of Reflection and feedback

### Theme 1- discrimination in service delivery

The experiences of medical students reveal a troubling reality within healthcare settings: discrimination in service delivery. One form of discrimination that they have observed is ageism, where older adult patients are often stereotyped and marginalized. These patients are sometimes seen as less important or less deserving of attention and care, which can lead to negative health outcomes. Another sub-theme that emerged from their accounts is the lack of cultural awareness or sensitivity among healthcare providers. This means that some providers may not understand or recognize the unique needs and perspectives of diverse patient populations, which can result in inadequate or inappropriate care. Furthermore, medical students have highlighted the issue of insufficient access to healthcare services for older people. Many older adults face barriers to accessing healthcare, such as transportation challenges or limited financial resources. These obstacles can prevent them from receiving the care they need. Lastly, medical students have noted the importance of adequate training for healthcare providers on how to address discrimination in medical settings. Without appropriate training and education, providers may unknowingly perpetuate discriminatory practices or fail to recognize signs of discrimination. Taken together, the narratives of these medical students underscore the need for healthcare systems to address discrimination and prioritize providing equitable care for all patients.

*“Sometimes, physicians attribute an older adult patient’s fatigue to “old age” or arthritis, without investigating whether there may be other underlying medical conditions or lifestyle factors contributing to the fatigue.” P 12*.

*“I observed some physicians did not take the time to listen to the older patient and understand their concerns. some physicians also use language that is condescending or dismissive, such as referring to the patient as “sweetie” or “dear,“ which can be patronizing and disrespectful.” P 04*.

To avoid this type of ageism and improve communication with older adult patients, physicians should approach each patient with an open mind and treat them as individuals with unique needs and concerns. They should take the time to listen attentively to the patient, ask questions, and conduct a thorough examination to ensure an accurate diagnosis and appropriate treatment plan. By doing so, physicians can establish trust and rapport with their older adult individuals, leading to better health outcomes and a more positive overall healthcare experience for both the physician and the patient.

### Theme 2- lack of inter-professional education

in this theme, we extracted that there is often insufficient collaboration between healthcare professionals when caring for older adult patients. This can result in fragmented care, with patients receiving different, sometimes conflicting, advice from different providers. To address these issues, our study highlights the need for more teamwork and leadership training in medical education. By promoting collaboration and effective communication among healthcare professionals, medical students can develop the skills they need to provide comprehensive, patient-centered care. Furthermore, providing opportunities for interdisciplinary education and training can help medical students gain a deeper understanding of the roles and perspectives of other healthcare professionals, which can improve coordination and quality of care for older adults.

*“I strongly advocate for a shift towards multidisciplinary collaboration in the field of medicine. This approach becomes even more crucial when dealing with older adult individuals, particularly those who are very old and may suffer from cognitive impairments that affect them not only physically but also psychologically. In addition to medical doctors, it is imperative to involve psychiatrists, physiotherapists, and other healthcare assistants to provide comprehensive care that takes into consideration the social factors impacting their health” P 09*.

*“As a medical doctor, I have witnessed firsthand the consequences of a lack of interprofessional collaboration and training in caring for older people. It is not uncommon for physicians to prescribe medications for older adult’s patients without consulting with a pharmacist to check for potential drug interactions or side effects. This can result in adverse reactions or complications that could have been avoided if there had been better communication between healthcare professionals from different disciplines.” P 01*.

the participants believe that the failure of healthcare professionals to work together effectively can lead to suboptimal outcomes for older patients, including medication errors, poor disease management, and insufficient support for their emotional and social well-being. It is therefore imperative that we prioritize interprofessional collaboration and training in caring for older adults to ensure that they receive holistic and comprehensive care.

### Theme 3- interpersonal communication skills

Interpersonal interactions and communication in healthcare delivery present several challenges, especially when it comes to older adult populations. The following sub-themes have been identified in the study as significant problems that healthcare providers are facing:

Communication barriers due to hearing or cognitive impairments in older adult patients: Healthcare providers face significant challenges when communicating with older person who have hearing or cognitive impairments. These communication barriers can lead to misunderstandings, misdiagnosis, and other negative outcomes. Addressing these barriers requires healthcare providers to develop effective communication strategies that overcome these challenges and ensure that patients receive proper care.

Challenges in addressing difficult or resistant patients and family members: Healthcare providers often encounter difficulties when dealing with patients and family members who are uncooperative or resistant to care. It is challenging to build trust and rapport with such patients, making it difficult to address their concerns and provide them with the right care they need. To overcome this challenge, healthcare providers must adopt a patient-centered approach to understand the patients’ perspectives and priorities.

Need for more patient-centered care approaches: Patient-centered care is critical in improving patient outcomes, satisfaction, and quality of life. However, healthcare providers may struggle to provide such care due to the diversity of health needs, preferences, and values among patients. Healthcare providers need to tailor their approach depending on the individual’s unique health needs and preferences to foster better relationships.

Importance of empathy and active listening skills for healthcare providers: Healthcare providers should show compassion towards their patients, understand their feelings, and listen actively to verbal and nonverbal cues. With these skills, healthcare providers can build trust and rapport with patients, leading to improved outcomes and patient satisfaction. However, healthcare providers may find it challenging to demonstrate empathy and active listening skills consistently due to competing demands and time constraints in healthcare settings.

*“Some older adult suffer from cognitive decline, which can affect their ability to communicate effectively with us. In such cases, we try to communicate with family or friends” P 03*.

*“Sometimes they talk so much that you miss the main clues, they really like to tell stories and they want someone to listen to their pain and heart (Laughing mode :-)”P 15*.

### Theme 4- limited knowledge, human resources, and physical infrastructure in older adult care

The provision of quality healthcare to older adult patients is a crucial issue, as the growing number of aging individuals requires specialized care. However, several sub-themes related to geriatric care have been identified that pose significant challenges to the delivery of high-quality care to older patients.

One such sub-theme is inadequate education and training in geriatric medicine among general physicians. Medical students may not receive enough specialized knowledge and training in geriatric medicine during their studies, leading to a lack of expertise in caring for older patients. This situation can result in suboptimal care and negative health outcomes for older adult patients.

Another sub-theme is the shortage of qualified healthcare professionals with expertise in geriatric care. With the demand for older adult care rising, there is a need for experienced and skilled professionals who can provide tailored care to older patients. However, there is a shortage of such professionals, which could lead to inadequate care for older patients.

The need for more resources and funding for older adult care is another sub-theme. Older adult care services require increased resources and funding to provide the best care possible to older patients. Without adequate resources, these services may not be able to provide the necessary medical equipment or staff to meet the complex needs of older adult.

Finally, the insufficient physical infrastructure to support older adult patients is a crucial sub-theme. Medical students may not get enough exposure to innovations that could enhance older adult care due to the lack of infrastructure to support older adult patients’ needs. Amenities must be designed to accommodate the unique needs of older people, including mobility issues, accessibility, and safety concerns.

In conclusion, addressing these sub-themes is critical to ensuring high-quality geriatric care for older adult patients. Efforts should be made to increase education and training in geriatric medicine, expand the pool of qualified healthcare professionals, provide more resources and funding for older adult care services, and develop infrastructure that supports the needs of older adult patients.

*“As a medical doctor student working in the internal medicine ward, I have observed a significant shortage of staff in the field of geriatric care. The small team of healthcare professionals who specialize in this area is overwhelmed by the high number of patients they see on a daily basis. I have seen them frequently work long hours to keep up with the demand, and it is clear that their workload is unsustainable.” P 02*.

*“An establishment that caters specifically to the needs of the older adult is necessary. It should be designed in such a way that it facilitates easy mobility for seniors and accounts for their physical limitations. As an intern, I struggled to navigate the hospital when I first started, so imagine how much more difficult it would be for an older person who requires a walker or other assistive device. Therefore, it’s crucial to have a capacity that promotes independence and ensures the safety of our older population.” P13*.

*“Despite their best efforts, some patients do not receive the quality of care they deserve due to the lack of resources available. It is disheartening to witness this situation, as these patients are often the most vulnerable and in need of specialized care. As a physician, I understand the importance of providing high-quality care to all patients, regardless of their age or medical condition. Therefore, it is essential that we address the shortage of staff and resources in the field of geriatric care to ensure that our patients receive the care they need and deserve.” P11*.

### Theme 5- enhancing attitudes towards older person care through experiential learning

Experiential learning is a type of learning that involves hands-on experiences, mentorship programs, community engagement, reflection, and feedback to help learners connect theory and practice. In the context of enhancing attitudes towards older person care, experiential learning activities can allow medical students to interact with older adults, learn about their experiences and needs, and develop empathy and compassion towards them.

Medical students believe that participating in a range of experiential learning activities can improve their understanding of the unique needs and perspectives of older adults. Additionally, clinical clerkships supervised by skilled mentors can help promote positive attitudes toward aging and challenge negative attitudes. This can involve evaluating the quality of training that students receive from their mentors or examining the impact of clinical settings on students’ attitudes toward caring for older adult patients.

one of the frequent codes for addressing negative attitudes was that Reflective practice involves the deliberate and structured process of thinking about and analyzing one’s experiences. In the context of medical education, reflective practices are to help medical students engage in critical thinking and self-reflection about their experiences with older adults and explore their own attitudes and biases towards aging and older adult care.

“*after interacting with an older patient, I often take the time to reflect on the experience. I think about what went well and what could have been improved, as well as any challenges or issues that arose during the interaction.” P 26*.

By engaging in community-based learning experiences, medical students have the opportunity to interact with older adults in a real-world setting, learn about their needs and experiences, and develop empathy and understanding toward them. community engagement includes volunteering at a nursing home, hospice, or senior center, or participating in community-based health initiatives for older adults.

Through these experiences, medical students can gain a deeper appreciation for the unique needs and challenges faced by older adults, such as chronic health conditions, social isolation, and mobility issues. Additionally, community-based learning experiences can help medical students develop important skills, such as communication, teamwork, and cultural competence, which are essential for providing quality care to older adults.

*“While there are many valuable experiences available through clinical rotations, such as interacting with older patients in hospital settings, these experiences may not provide a comprehensive understanding of the unique needs and challenges faced by older adults in the community. We may miss out on important opportunities to learn about the social and cultural factors that can impact older adults’ health outcomes, as well as the challenges faced by older adults in accessing healthcare services.” P 19*.

### Discussion and conclusion

In this study, we explored the attitudes of general medical students towards older adult care in a lower middle-income country and identified several root factors that impact these attitudes. The four main themes we identified were discrimination in service delivery, a lack of interprofessional training, interpersonal interactions and communication, and inadequate infrastructure and human resources.

Discrimination in service delivery, this theme shows that medical students may discriminate against elderly patients, leading to suboptimal care. Our findings align with previous research on ageism in healthcare, which has shown that negative attitudes and stereotypes toward older adults can lead to discriminatory practices and poorer health outcomes [5]. The systematic review by Liu [10] found that nurses generally hold positive attitudes towards older people, but there were some negative attitudes related to communication and providing care for patients with dementia or cognitive impairment. The authors identified a need for further research and education to improve nursing care for older adults. The authors note that negative stereotypes and biases about older adults are pervasive and can lead to poor healthcare outcomes for this population. Discriminatory practices, such as denying treatments or procedures based on age, may also be present in healthcare settings. They discuss addressing ageism in healthcare requires a multifaceted approach. Healthcare providers should receive education and training on how to recognize and combat ageism, while policy changes and advocacy efforts can help to eliminate discriminatory practices. Ultimately, eliminating ageism within healthcare is essential for ensuring high-quality care for all patients, regardless of their age [6].

The second theme was a lack of interprofessional training, which highlights that medical students may not receive adequate education on how to work effectively with other healthcare professionals in caring for older adult patients. This finding is consistent with previous research showing that interprofessional collaboration is essential for high-quality care for older adults [11]. One study found that interprofessional education improved knowledge, attitudes, and skills related to geriatric care among medical, nursing, and pharmacy students [12]. Another study found that interprofessional teamwork reduced medication errors and improved patient safety in a geriatric ward [13].

The third theme was interpersonal interactions and communication, which illustrates that medical students struggle to communicate effectively with older adult patients. This finding is supported by previous research showing that poor communication between healthcare providers and older patients can lead to misunderstandings, decreased satisfaction, and worse health outcomes [14]. One study found that training in communication skills improved medical students’ ability to communicate with older patients and their families [15]. Another study found that a communication skills training program for hospital staff improved patient-centered communication and reduced patient anxiety [16].

The fourth theme was limited physical infrastructure and human resources, which shows that medical students may struggle to provide high-quality care for older adult patients due to limitations in healthcare resources. This finding is consistent with previous research showing that healthcare systems often do not have the necessary resources to meet the needs of aging populations [17]. One study found that staffing shortages in nursing homes led to decreased quality of care and increased risk of adverse events [18]. A study by Feng highlights the importance of developing a comprehensive and integrated long-term care system that can effectively provide care and support for China’s aging population [19].

Overall, our study provides important insights into the attitudes of medical students toward older adults care in a lower middle-income country. based on the findings of your study, there are several implications for medical education policy makers.

Firstly, it is important to incorporate training programs that address ageism and its impact on healthcare delivery as part of the medical curriculum. This can be achieved through the development of interprofessional education programs that promote collaboration among healthcare professionals from different disciplines. Furthermore, communication skills training should be incorporated into the medical curriculum to enhance students’ ability to communicate effectively with older adults and their families.

Secondly, policy makers should aim to improve the infrastructure and human resources available to healthcare systems to provide better care for older adult patients. This can be achieved through increased investment in geriatric healthcare services, staffing levels, and home-based care services.

Thirdly, medical education policy makers should promote research and evaluation of healthcare systems that focus on aging populations. By studying the unique challenges faced by older adult patients and identifying effective strategies to address these challenges, healthcare providers can offer more tailored and effective care.

Finally, incorporating experiential learning opportunities for medical students, such as clinical placements in geriatric care settings, can help to promote positive attitudes towards older adult and increase awareness of the importance of providing high-quality care to this population. in this regard, a systematic review examined the effectiveness of various educational interventions on healthcare student behaviors and attitudes toward older people. The most common type of educational intervention identified was interaction with real patients, and interventions that incorporated interactions with independently living older patients were found to have a positive impact on student attitudes toward older adults [20].

In summary, medical education policymakers should prioritize efforts to reduce ageism and improve healthcare delivery for older adult. By implementing targeted training programs, investing in healthcare infrastructure, promoting research and evaluation, and offering experiential learning opportunities, medical education policymakers can play a crucial role in ensuring that future healthcare providers are equipped to provide high-quality care to aging populations.

### Limitations

One of the most significant limitations was the small sample size that we used. Although we obtained data saturation from the 27 participants who took part in our qualitative study, this number may still be considered limited, and it is possible that if more participants had been included, additional themes or perspectives might have emerged. Another limitation of our study concerns its generalizability. The research was conducted exclusively with medical students from a university located in Iran. As such, our findings may not necessarily apply to other populations or contexts, which limits their broader relevance and applicability. Finally, we recognize that participants may have been influenced by social desirability bias when responding to our questions. This means that they may have provided answers that they believed were socially acceptable or desirable, rather than reflecting their true attitudes or experiences. While we tried to account for this potential bias during our analysis, there may still be some limitations to the validity of our results as a result of this influence.

## Data Availability

The content of the semi-structures interviews analyzed in this study is available from the corresponding author on reasonable request.
